# Examining the nexus between medical education and complexity: a systematic review to inform practice and research

**DOI:** 10.1186/s12909-023-04471-2

**Published:** 2023-07-05

**Authors:** Kathryn Ogden, Sue Kilpatrick, Shandell Elmer

**Affiliations:** 1 Tasmanian School of Medicine, University of Tasmania, Launceston, TAS Australia; 2 Launceston Clinical School, Locked Bag 1377, Launceston, 7250 Australia; 3 School of Education, University of Tasmania, Launceston, TAS Australia; 4 School of Nursing, University of Tasmania, Launceston, TAS Australia

**Keywords:** Medical Education, Complexity Theory, Complexity Science, Literature Review, Curriculum, Evaluation, Research

## Abstract

**Background:**

Medical education is a multifarious endeavour integrating a range of pedagogies and philosophies. Complexity as a science or theory (‘complexity’) signals a move away from a reductionist paradigm to one which appreciates that interactions in multi-component systems, such as healthcare systems, can result in adaptive and emergent outcomes. This examination of the nexus between medical education and complexity theory aims to discover ways that complexity theory can inform medical education and medical education research.

**Methods:**

A structured literature review was conducted to examine the nexus between medical education and complexity; 5 databases were searched using relevant terms. Papers were included if they engaged fully with complexity as a science or theory and were significantly focused on medical education. All types of papers were included, including conceptual papers (e.g. opinion and theoretical discussions), case studies, program evaluations and empirical research. A narrative and thematic synthesis was undertaken to create a deep understanding of the use of complexity in medical education.

**Results:**

Eighty-three papers were included; the majority were conceptual papers. The context and theoretical underpinnings of complexity as a relevant theory for medical education were identified. Bibliographic and temporal observations were noted regarding the entry of complexity into medical education. Complexity was relied upon as a theoretical framework for empirical studies covering a variety of elements within medical education including: knowledge and learning theories; curricular, program and faculty development; program evaluation and medical education research; assessment and admissions; professionalism and leadership; and learning for systems, about systems and in systems.

**Discussion:**

There is a call for greater use of theory by medical educators. Complexity within medical education is established, although not widespread. Individualistic cultures of medicine and comfort with reductionist epistemologies challenges its introduction. However, complexity was found to be a useful theory across a range of areas by a limited number of authors and is increasingly used by medical educators and medical education researchers. This review has further conceptualized how complexity is being used to support medical education and medical education research.

**Conclusion:**

This literature review can assist in understanding how complexity can be useful in medical educationalists' practice.

**Supplementary Information:**

The online version contains supplementary material available at 10.1186/s12909-023-04471-2.

## Background

* “Medical education is a busy, clamorous place, where a host of pedagogical practices, educational philosophies, and conceptual frameworks collide” *[1]

Medical education straddles the university, healthcare and community sectors, and brings together a wide range of pedagogies and philosophies [1] in an attempt to create a workforce that possesses a broad range of competencies and capabilities, spanning specialities as diverse as, for example, general practice, neurosurgery, radiation oncology, psychiatry, pathology, cardiology and public health. The evolution of medical education has occurred across centuries and seen many innovations and paradigmatic shifts [2–4]. Medical education in its full depth and breadth in the 21^st^ Century is a multifarious endeavour. The use of complexity, as a theory or science, to aid this understanding is entering the literature, however the extent that it is used and subject matter that it addresses has not been scrutinised. Discovering how complexity is being used to support the development and understanding of medical education can inform others in developing and researching medical education programs.

Complexity as a scientific paradigm gained momentum and widespread attention from a movement of the 1970s and 80s when an interprofessional group of scientists sought to develop a collective understanding of phenomena that were non-linear in nature and represented the dynamics of real-world systems [5]. This was a move away from the reductionist scientific paradigm that dominated the previous centuries. The science of complexity saw a shift in thinking towards an appreciation that the whole cannot always be explained by examining its individual components. Central to complexity are complex adaptive systems which are comprised of many individual components or agents that behave and interact according to a set of rules including responding to feedback from other agents in the system. The interaction and adaptation of these agents results in dynamic and self-organising properties with the emergence of a global structure or system [5, 6].

Complexity is increasingly used to help understand social systems [7, 8], including health care and educational systems [9]. Central to this is understanding health care as a series of interdependent complex adaptive systems [10]. With this in mind, this literature review explores the extent and nature of the use of complexity in medical education. In doing so it will assist those involved in medical education and medical education research to determine the relevance of complexity to their practice.

To a novice in the field, the issue of terminology is confusing, yet important to tackle. There are various terms used to indicate what is apparently the same field including ‘complexity science’, ‘complexity theory’, ‘complexity research’, ‘complex systems research’, ‘the science of complexity’ and for some simply ‘complexity’. The different terms may indicate a different orientation of complexity, however they can be considered under a single umbrella [11], and for the purposes of this paper the term ‘complexity’ will be used.

Distinguishing complexity from complicatedness is also crucial. Complicated systems can be understood as the sum of the individual elements, however a complex system is characterised by the presence of interacting parts which result in a collective, emergent outcome unable to be understood by examination of its individual components [7]. There are many orientations and approaches to complexity. Morin [12] and Manson [13] provide detailed distinctions between the orientations and Castellani [11, 14] describes them as a collection of innovations that have been hailed a “revolution in thinking – a paradigm shift” [14] that aim to address the questions of a globalised society with complex social problems.

Academics who propose complexity as a theory for education identify learning as a complex process which occurs in complex systems, leading to the emergency of knowledge in the context of educational systems which, when optimised, are adaptive, resilient, responsive, and networked within and aside other systems.[15–19]. Castellani and Gerrits identify education, health care, and public health as content topics within the systems theory and complex systems theory traditions [11]. Complexity as a frame of reference for education allows a reimagining of education that is less didactic and more transformative with important links to previous scholars such as Dewey [19] and Vygotsky [20]. Alhadeff-Jones identifies complexity as providing assumptions and principles that challenge dominant systems of thought [15] and Doll points to learning that is not through direct transmission from expert to novice, but non-linear with multiple perspectives being shared and a curriculum that is “open, dynamic, relational, creative, and systems oriented” [19]. Davis and Sumara argue that complexity could be construed as an educational theory [17] and Byrne argues for the integration of a complexity frame of reference into curricula broadly [16]. Mason notes that educational research informed by complexity asks different questions, avoids an input–output ‘black-box’ causal modelling, and that curriculum and teaching can be understood as emergent phenomenon [21]. This review aims to develop an understanding of how complexity is, and can, unlock the black box specifically in medical education.

## Methods

This literature review does not fit neatly into a typical typology of reviews. An initial scoping of the literature identified the need for a method to integrate publications that were opinion and commentary, conceptual and theory building, and empirical studies that used complexity as a theoretical lens. A meta-narrative review methodology [22] was appealing because of its grounding in the work of Thomas Kuhn, which recognises that science undergoes paradigmatic shifts when current theories cannot explain phenomenon and a new theory is proposed.. This resonated with the purpose of the review however meta-narrative reviews aim to explore a range of research traditions on a topic [22], which fails to describe this review with its focus on the impact that a single epistemological approach (complexity) has had on a discipline of practice and research (medical education). The guiding principles of a meta-narrative review remained as a framework for how the review was conducted. This review, therefore, is a narrative review which takes a systematic approach, incorporating the guiding principles of a meta-narrative review.

The aim of the review is to build a rich picture of the position of complexity in medical education through the integration of all publications that engage in both. To do so requires building both a narrative of how complexity is inserted into medical education, in addition to thematically examining the facets of medical education where authors have either theorised about or utilised complexity. The six guiding principles of a meta-narrative review are: pragmatism, pluralism, historicity, contestation, reflexivity and peer review [23]. See the Additional Materials 1 for a discussion of these principles in the context of this review. Through reflexivity and peer review we determined that the principle of pluralism with respect to inclusion of all research traditions, a necessary ingredient for meta-narrative, was counter to the aim of the review whose focus is a single epistemological approach. We therefore took a pragmatic approach to the review. Given the review incorporates conceptual papers and is not focused entirely on empirical research it was not eligible for inclusion in the International Prospective Register for Systematic Reviews, Prospero [24].

**Fig. 1:**
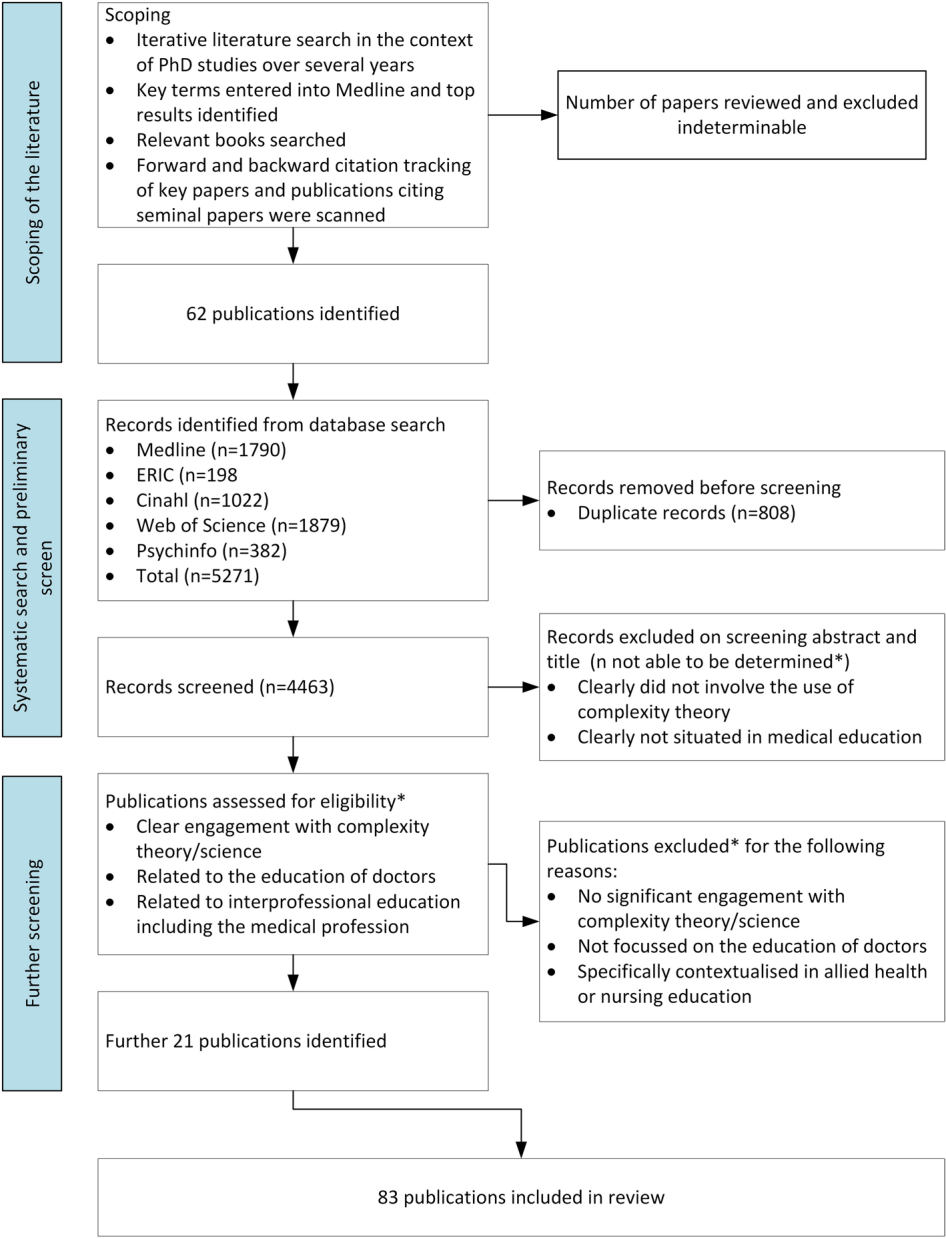
Modified PRISMA diagram representing the search processes

The identification of relevant literature began as a scoping exercise in 2015. Medline was searched iteratively using relevant search terms, with reference lists of relevant papers explored for further publications. Forward and backward citation tracking was made of key papers and publications citing seminal papers were scanned. Books which related to medical education or complexity were examined for relevant chapters. Over this time the authors became familiar with the use of complexity in healthc are and the broader literature. In November 2022, a systematic search of the literature was undertaken using five databases to locate papers not yet identified (Table 1). Search processes and outcomes are provided as a modified PRISMA diagram in Fig. 1.

**Table 1:** Databases and search terms

Database	Search terms
Medline	"Education, Medical"[Mesh] AND ((complexity theor*) OR (Complexity science)) *n* = 1,790
ERIC	(complexity theory OR complexity OR complexity science OR complex adaptive systems OR complexity approach) AND (medical school OR medical education OR medical students OR medical curriculum OR medical student education OR clinical education) *n* = 198
Cinahl	(complexity theory OR complexity OR complexity science OR complex adaptive systems OR complexity approach) AND (medical school OR medical education OR medical students OR medical curriculum OR medical student education OR clinical education) *n* = 1022
Web of science	((("complexity theory") OR (complexity) OR ("complexity science") OR ("complex adaptive systems") OR ("complexity approach")) AND ("medical school") OR ("medical education") OR ("medical students") OR ("medical curriculum") OR ("medical student education") OR ("clinical education"))) *n* = 1,879
Psychinfo	complexity.mp. and exp Medical Education/ *n* = 382

All types of publications were potentially included – including theoretical papers, letters, opinion/commentary pieces, and editorials. Publications were included if they genuinely engaged with complexity, and this was in one of three ways:

1. The primary purpose of the publication is to advance the understanding of complexity in the context of medical education.
2. Complexity is used instrumentally in building commentary or theory, or to support empirical research, but not the primary focus.
3. Complexity is an important aspect of the commentary or theory, or is used as a theoretical lens for empirical research, but is not central to it.

Publications also were included if they related significantly to the education of doctors (including interprofessional education), including undergraduate, post-graduate and ongoing education. Papers were excluded if there was only a passing or no mention of complexity as a theory or science or if education of doctors was not a significant focus. No further limits were applied. Titles and abstracts were scanned for potential suitability. Full text was retrieved for all studies that were potentially suitable and a final decision on inclusion made according to the criteria described. This process was undertaken by a single researcher (KO) overseen and reviewed by the two co-authors. A limitation is that the process is not likely to be replicable, however the purpose of the review is not outcome based, rather it is a descriptive analysis of a body of theoretical and empirical literature at a point in time. As with qualitative research, the results are impacted by the experience and expertise of the primary author who has developed a deep understanding of the specific and broader literature over many years.

Bibliographic information was collected including author name, year of publication, and publication journal. Only qualitative data were extracted as none of the included empirical publications used quantitative measures. All publications were reviewed in their entirety and a summary made. Summaries were then examined, and the themes incorporated within each publication were identified. Overarching themes were identified, and publications sorted accordingly, with the majority (50/83) addressing more than one theme (see Additional Materials 2 for a full list of publications mapped to themes, type of publication and type of engagement with complexity). Main messages were extracted from each summary, and these were integrated across each theme to provide narratives and summaries of thematic groupings relating to the use of complexity in the medical education academic literature. Risk of bias assessment was not deemed necessary to achieve the objectives of the study.

## Results

### Bibliographic information

**Fig. 2:**
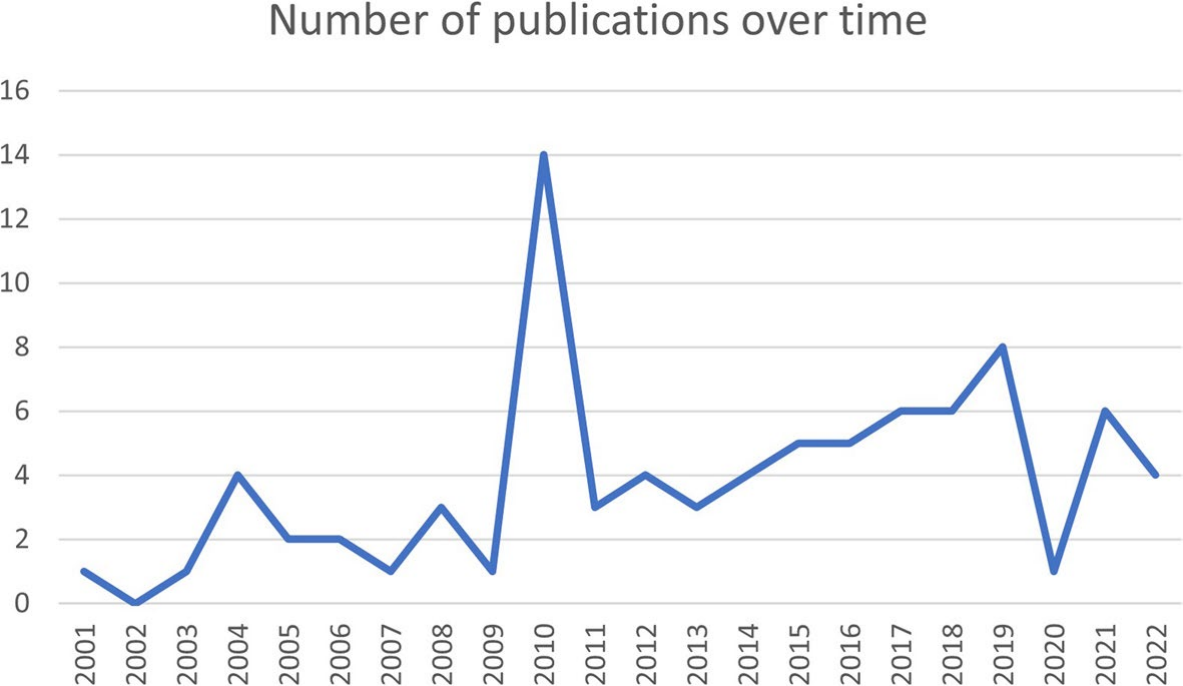
Number of publications over time: Nexus between complexity and medical education

Eighty-three publications were included [18, 25–106], 17 were commentaries, 46 were conceptual, 19 reported on empirical studies the majority of which were case studies and program evaluations. There were 57 individual primary authors, 15 of these were the primary author for more than one publication accounting for almost half of the publications (49.4%). The majority of publications were from English speaking countries, with the USA, Canada and the UK accounting for more than 25% each (Table 2). The number of publications by year are shown in Fig. 2. There was a gradual increase from 2001, with a spike in 2010. There was a decline in 2019; and evidence from 2021 and 2022 (although incomplete) that this is reversing. There were 28 different sources, 25 journal articles and three book chapters. Six journals had five or more publications, these were Academic Medicine, BMC Medical Education, Journal of Interprofessional Care, Journal of Evaluation in Clinical Practice, Medical Education and Medical Teacher.

**Table 2:** Characteristics of included studies

Characteristic	Number of papers n (% of total)
**Methodology**	
Commentary	17 (20.5)
Conceptual	46 (55.4)
Empirical	19 (22.9)
Case study	9
Program evaluation	6
Qualitative study	2
Assessment of an intervention	1
Participatory action research	1
Literature review	1 (1.2)
**Country**	
USA	27 (32.5)
UK	22 (26.5)
Canada	21 (25.3)
Australia	8 (9.6)
Netherlands	2 (2.4)
Ireland	1 (1.2)
South Africa	1 (1.2)
**Number publications per primary author**	
Primary author has 1 publication	42 (50.6)
Primary author has > 1 publications	41 (49.4)

### Overview

**Fig. 3:**
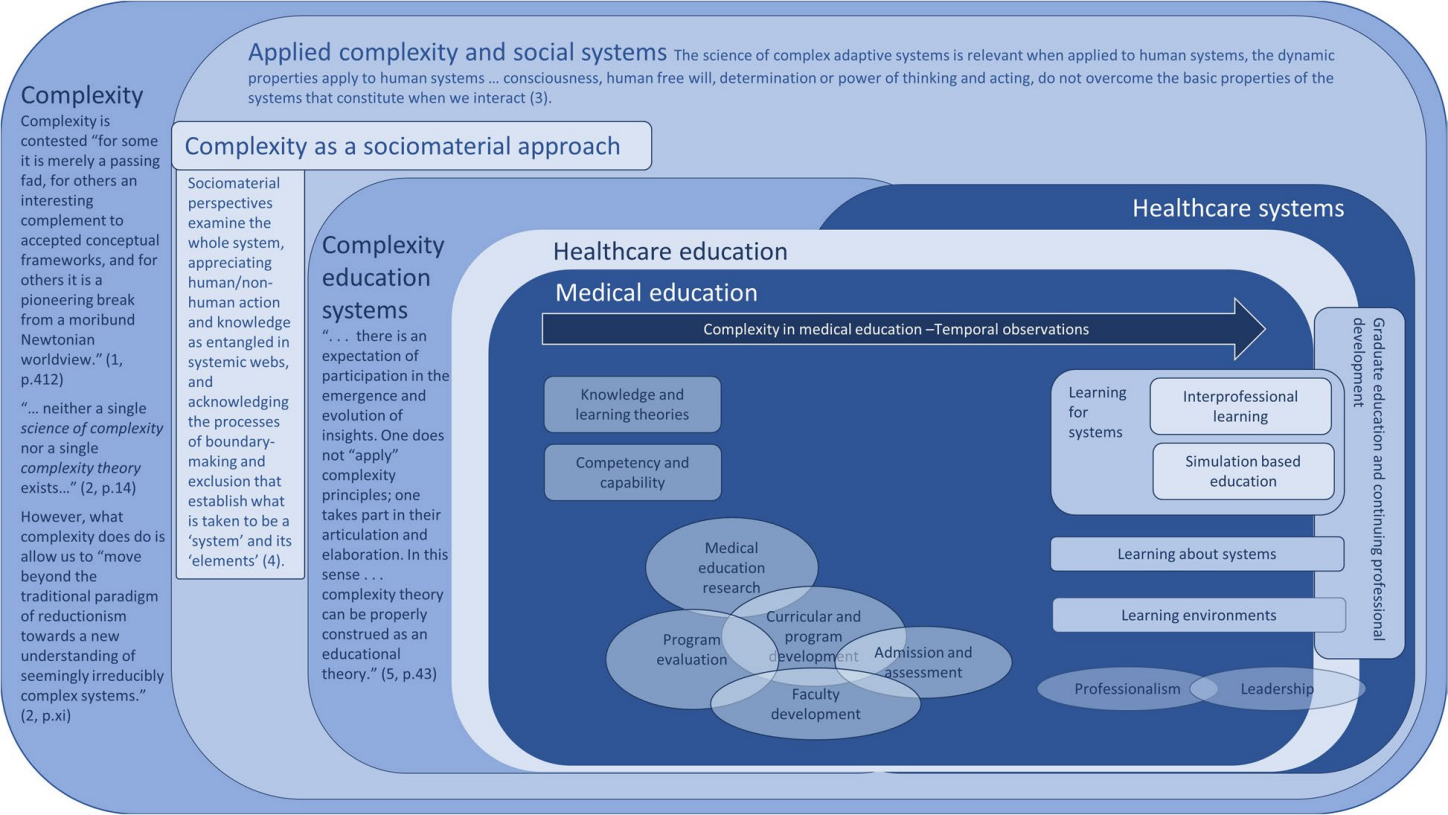
Narratives of complexity and medical education. ^1^Manson SM: Simplifying complexity: a review of complexity theory. Geoforum 2001, 32(3):405–414. ^2^Mitchell M: Complexity: A guided tour. New York. NY: Oxford University Press; 2009. ^3^Stacey R: Emerging strategies for a chaotic environment. Long Range Plann 1996, 29(2):182–189. ^4^Fenwick T, Nerland M, Jensen K: Sociomaterial approaches to conceptualising professional learning and practice. Journal of Education and Work 2012, 25(1):1–13. ^5^Davis B, Sumara D: Complexity as a theory of education. TCI (Transnational Curriculum Inquiry) 2009, 5(2):33–44

The story of the temporal and spatial insertion and evolution of complexity in the medical education literature is explored, followed by a discussion of what the reviewed literature has to say directly about the purposes, value and relationship of complexity to medical education. The relationship between complexity and socio-materiality is discussed, given socio-materiality was frequently incorporated into writings. From here, a thematic analysis is provided of seven specific aspects of medical education where complexity was seen as pertinent, or complexity was used as a theoretical lens. These are: competency and capability, knowledge and learning theories; curricular, program and faculty development; program evaluation and medical education research; assessment and admissions; professionalism and leadership; and learning for systems, about systems and in systems. Figure 3 diagrammatically represents the findings.

### The insertion of complexity into medical education

While there is evidence that the use of complexity as a theory for medical education is becoming established, to date most publications come from English speaking countries, with 84% from the USA, Canada, and the UK, and a further 10% from Australia. Almost half (41) publications have primary authors who have contributed more than one publication; there are 15 such authors. Complexity is yet to appear in the literature outside western populations and there are a small number of authors who contribute to a significant proportion of the literature (Table 1).

Early publications sought to establish complexity as a relevant theory, and then link complexity to knowledge and learning theories, and competency and capability. More recent publications use complexity to explore curricular development, and as an explanatory or exploratory lens to examine more specific aspects of medical education. This is demonstrated in Fig. 2 whereby the early literature is represented in the outer layers of the (square) onion; over time publications focused on more specific aspects of medical education represented at the right of in the inner core.

One publication not formally included in the review but worthy of reference is an invited presentation to the 100^th^ meeting of the Association of American Medical Colleges [107]. While the essay does not explicitly link to complexity, which was taking hold at the time through the work of the Santa Fe Institute, metaphors of a musical symphony resonate with complexity, including a conclusion that the musical metaphor was chosen because “each level of musical organisation has emergent properties that are not predictable from the simpler” [107]. One included study picks up on the same musical metaphor [79].

A decade later complexity was introduced to the health care profession in a series of four papers published in the BMJ [54, 108–110], one of which specifically focused on education [54]. This has become a sentinel publication and was followed by an increase in the number of publications per year, with a significant spike in 2010 (Fig. 2). This spike is partly explained by an edition of the Journal of Evaluation in Clinical Practice dedicated to complexity and health professions education, with an editorial that notes medical education would be better served by contemporary learning theories and concepts than the reductionist explanations traditionally employed [80]. Five of the included articles come from this edition [18, 30, 45, 80, 97]. The other occurrence in 2010 was the 100-year anniversary of The Flexner Report [111], with two included papers reflecting on the next 100 years in the context of complexity [68, 81].

2011 saw a challenge to the way complexity was being used in the medical education literature, in particular its adoption at the apparent expense of linear research methodologies [84]. Norman argued that when taking into account the true origins and definitions of chaotic and complex systems, medical education does not conform [84]. This is because complex and chaotic systems have characteristics that are not likely to be present in educational systems, and linear approaches have provided useful insights. Regehr responded, noting that there is not a call to stop using experimental methodologies but rather their misuse, and maintaining that while there is a danger in going too far with the metaphors employed, complexity is valuable in that it provides an additional way of thinking that does not reduce intervention to linear parts [89]. What appears to be at the heart of the disagreement is the orientation of complexity used by the respective authors. This was elaborated by Cristancho et al. who reviewed the manner in which medical education was employing complexity, with concern that scholars did not understand the multiple orientations, highlighting how this can be problematic, and encouraging constructive dialogue acknowledging the multiple orientations [42]. Several valid frameworks for the multiple orientations of complexity are identified by Cristancho et al. [41] but Manson’s [13] framework is specifically noted. It identifies approaches to complexity science based on discipline, these are: algorithmic whose origins are in mathematics; deterministic whose origins are in physics; and aggregate whose origins are in biology [13]. All share a concern for the nature of systems which are non-reductionist. Bleakley also acknowledges the “academic spat” between those who object to the broader, more liberal interpretation of complexity and academics like himself who used a wider application of complexity which sees a conversion to holistic models such as systems, chaos and network theories [30]. A similar argument has played out in the health care literature [108, 109, 112–114].

A downturn in publications in 2020–2021 could be explained by the COVID-19 pandemic diverting the efforts of the medical education community towards issues relevant to adaptation to the pandemic. One recent paper relates complexity and medical education within the context of the COVID-19 pandemic [85].

### Complexity as a theoretical lens for medical education

Doll Jr and Trueit provide an historical context of medical education and its transition from an Aristotelian/Ptolemaic view of the world to a Copernican/Newtonian one, and in modern days a further paradigmatic shift which recognises the experimental tradition as “excluding too much of the messiness, uniqueness, and vitality of life” [45]. Davis and Sumara agree and argue that there is a compelling mass of evidence against Euclidean and Newtonian assumptions of popular educational theories and the contribution of complexity is in demonstrating that many phenomena can only be understood at the level of emergence [18]. Linear reductionist approaches to health profession education are challenged [80] and complexity identified as providing new ways to study education systems [49, 81]. The conceptualisation of nested learning adaptive systems encourages rethinking of the pragmatics of teaching [18], with its focus on interconnections between people and systems key to complexity’s usefulness [64].

According to Mennin, the challenges of medical education will require paradigmatic, not incremental, change, for which complexity offers a way to consider [82]. Applied complexity is identified as the branch that relates to health care and education [11], and while it is not a panacea, complexity can provide a number of important tools for inquiry into social complexity [14]. However, in exploring the usefulness of complexity for examining professional learning in collaboration Fenwick urges caution in the translation of complexity into the domain of human activity given its origins in mathematical and ecological systems [49]. This aligns with Paley in challenging those who have utilised it more liberally [115]. These authors urge against the use of metaphors and romanticism in the use of complexity, encouraging a deep analysis of the intricate sociomaterial dynamics of emergence.

### Sociomateriality and complexity

Authors in this review who cite Sociomateriality identify complexity as a sociomaterial approach [51, 71, 72, 75, 78, 101], as guided by the writings of Fenwick [49, 50] who notes that complexity as it relates to social systems has shared premises with Sociomateriality. Sociomateriality is an approach to organisational studies that “posits the constitutive entanglement of the social and the material in everyday life” [116] promoting the view that the technical and social aspects of work are inherently inseparable. Sociomateriality refers to theoretical approaches which move away from the human as the central focus towards an exploration of the non-linear relationships between materials and social practices. In a practical sense the social and material combine to shape and enact professional practice [58]. Complexity is identified as one of many theoretical approaches aligned with Sociomateriality, all with differing origins but a common focus on non-linear relationships between materials and social practices [49–51, 71, 72, 75, 78, 101]. Sociomateriality and complexity both emphasise the importance of understanding systems as a whole – the interconnectedness of material conditions with social relationships in the case of Sociomateriality and the interactions of components of a system that give rise to emergent properties in the case of complexity. They provide complementary perspectives on the importance of considering the interconnectedness within and between social systems.

It is argued that approaches to research which are informed by Sociomateriality allow the recognition and investigation of “multiple, emergent, and shifting Sociomaterial assemblages that constitute organisations” [116]. Sociomaterial perspectives are relevant in opening methods that make visible the messy dynamics of professional learning [117]. Limited attention is paid to the importance of materiality in medical education, with a human-centric view that fails to note relations among social and material forces, and conflict between ideals of evidence-based models and sociomaterial contingencies of clinical practice [50].

Fenwick [50] and Goldszmidt [58] further explore the value of sociomaterial approaches in medical education. Drawing on a description of a busy internal medicine ward, Goldszmidt demonstrates how the social and material are woven into the everyday work of the team, and such a lens can enable new questions and ways of exploring existing problems [58]. Fenwick identifies three concerns when considering medical practice and learning: that there is limited attention paid to the importance of materiality; that a human-centric view fails to note relations among social and material forces; and that there is conflict between ideals of evidence-based models and sociomaterial contingencies of clinical practice. A sociomaterial approach to learning embraces a range of theories that share social and material forces, culture, nature, and technology as enmeshed in everyday practice [50]. In short, for medical education, sociomaterial approaches place importance on the relationship between social and physical environments and how these contribute to learning [78].

### Competency and capability

In their landmark paper, Fraser and Greenhalgh note that complexity thinking challenges medical educators to educate for capability, “the ability to adapt to change, generate new knowledge, and continuously improve performance” [54] and identify pedagogical approaches that can be used to achieve capability. Rees and Richards further argue that if educators themselves were comfortable with complexity it would help develop capability as educators [87]. Aron [25] and Cooper and Geyer [38] distinguish between competency and capability with competency achieved through knowledge of basic science, and a requirement for capability. The integration of basic science into clinical experience is established in medical education, however it is argued that translation into challenging clinical environments requires capability [46], recognising learning as an emergent process [25, 38, 46].

Dornan et al. distinguish capability from competence as an integration of knowledge, skills, personal qualities and understanding into clinical practice, something which requires experience in practice and which competency-based medical education frameworks do not account for [46]. Batt et al., also argue that competency frameworks in health care education can be improved to address contemporary practice through employing a systems thinking framework that incorporates complexity to make visible features of clinical practice otherwise overlooked [27]. The notion that capability relates to ability to integrate skills into complex clinical environments is supported by Fenwick and Dahlgren who caution that complexity itself does not distinguish between competency and capability; that the constructs of complexity explain systemic capabilities, not individual performance [51].

### Knowledge and learning theories

Complexity provides a lens for how knowledge is gained and how it relates to traditional theories of learning [29, 30, 32, 34, 44, 48, 50, 92, 95, 98]. Bleakley’s stated liberal line of complexity aligns with contemporary social learning theories which explain how learning occurs through collaborative education, rather than more dominant theories that focus on individual learning [30]. Authors in this review identify a wide range of learning theories that align with complexity, including script theory, assimilation theory, self-regulated learning and situated cognition [48]; Paragogy and Heutagogy [34]; Dreyfus Model and Satir Model [44]; problem-based learning [44, 79]; and Communities of Practice, Reflective Learning and Transformative Learning [92]**.** This diversity is consistent with Davis and Sumara’s view that complexity has emerged as an interdiscourse, that it arises in conversations of multiple diverse perspectives and is attentive to the “rhetoric of listening, participating and engaging” [18].

Employing the tradition of ‘Ockham’s razor’ [118], Bleakley illustrates the reductive nature of medicine, noting that medical educators are primarily a clinical and not academic body whose dominant cultural and scientific paradigm is reductive, impeding the wide adoption of complexity aligned social learning theories [30]. It is acknowledged that the application of complexity to the social and cultural spheres of human life is contested, however suggested that the explicit liberal line of complexity, with its wider application, aligns complexity with social learning theories to better understand medical education [30].

### Curricular, program and faculty development

Complexity is seen as a transformative theory of education by Davis and Sumara [18], Doll Jnr and Trueit [45], and Fenwick and Abrandt [51], mostly educationalists whose experience lies outside medical education. Development, if not transformation, was a strong theme among the reviewed literature relating to curricular, program and faculty development.

Complexity as a theoretical lens for program and curricular development was widespread [25, 38, 40, 44, 53, 57, 71, 73, 81, 105]. Traditional reductionist approaches in medical education were deemed necessary but not sufficient to both explain phenomena in medical education [25, 80], and provide a framework for evaluation and improvement [40, 55, 73, 81]. Guidance for curricular design, development and revision using complexity principles was provided [57, 73, 81], with some authors demonstrating tangible examples of curricular development processes in the areas of interprofessional education [38, 40, 105], integration of basic and clinical sciences [25] and educational and clinical services [44], and competency based medical education [53].

In introducing medical educators to relevant key concepts of complexity, Mennin aimed to stimulate discussion that would review and reframe approaches to the integration of curricula and the teaching–learning dynamic fundamental to medical education [81]. The curriculum planning process is described as a complex adaptive system, nested within other complex adaptive systems. Self-organisation is highlighted as a bottom-up approach for curricular development and reform, where interactive curricula lead to recursive interactions, greater disturbance, and more likelihood of self-organisation. Integration with top-down curriculum planning is acknowledged as complementary [81].

Goldman and Mintz describe how principles of complexity guided the process of curricular revision within a framework of shared leadership, understanding change as emergence and encouraging self-organisation, simple rules and constant principles for coherence over time, and continuous adaptation [57]. Complexity can act as a unifying theory and make a connection between the art and science of medical practice [25].

Four publications [52, 70, 87, 94] specifically address faculty development in medical education with a complexity lens, with it being a secondary focus for others [40, 44, 57, 74]. An example of a leadership program for course coordinators (as middle managers), informed by complex adaptive systems theory, enabled a whole of system, non-hierarchical approach to leadership and management which overcame reluctance to embrace leadership roles [70]. Capacity building in faculty development for clinical educators was informed by a systems perspective of learning, cognition, expertise and capability, with faculty development attending to capabilities required to work and innovate within complex systems [94] and to conceptualise how clinical educators could play a role as change agents [74]. Complexity theory is applied to the evaluation of faculty development, situating it as a dynamic process that evolves over time and for which a complexity lens considers multiple and unpredictable outcomes and impacts [52].

### Program evaluation and medical education research

A complexity frame for medical education research challenges the traditional tendency of the profession to feel most comfortable with reductionist methodologies [33, 38, 88]. It is suggested that medical education research should not be about finding generalisable solutions, but rather creating and sharing better ways of thinking about local problems to better generate rich understandings of the complex environments in which medical education is uniquely embedded [88].

The importance of using theory to determine evaluation approaches is highlighted, and evaluation frameworks informed by complexity are identified as enabling continual improvement in medical education programs [55, 72, 90]. Complexity requires moving beyond evaluation as outcome only, and to understand medical education as dynamic processes made up the diverse components and their interactions [55], with multiple and varied needs in a constant state of change [38]. Logic models and sensemaking are identified as useful in designing evaluation frameworks which incorporate complexity thinking [72].

However complexity is not a research method itself, rather it provides a ‘synthesising structure’ and it is still up to researchers to identify methods and methodologies from within the structure of a complexity approach [33]. Medical education researchers are urged to consider novel ways to present results, for example by considering the value of narrative in representing complexity [41], taking learnings from research methods in systems engineering [90], and the use of rich pictures [43].

### Assessment and admissions

It is argued by Durning et al. that while clinical reasoning is a non-linear process, traditional assessment strategies assume linearity of medical competence [48]. Complementary approaches grounded in the concept of non-linearity are proposed such as script concordance testing, concept mapping, microanalytic assessment of self-regulated learning (elaborated on elsewhere [119]), and workplace-based assessment [48]. The notion of borderline competence is identified as a zone that can be conceptualized as a non-linear three-dimensional framework that takes into account both clinical significance and performance difficulty [97]. The implementation of programmatic assessment is analysed with complexity identified as a relevant theoretical lens [102]. Complexity also provides an epistemology for understanding and analyzing a method of assessment for selection into medicine that promotes approaches that adapt and emerge, enabling widening access among under-represented groups [36].

### Professionalism and leadership

The narratives of professionalism and leadership address the intersection between medical education and healthcare systems. Hafferty identifies six waves of the modern medical professionalism movement [67]. The fifth wave links structure and agency and proposes a focus on a view of professionalism that moves from changing individuals to modifying structural and environmental forces. More nascent is the sixth phase which draws on complexity and recasts “social actors, social structures, and environmental factors as interactive, adaptive, and interdependent” [67]. Hafferty and Castellani track the development of medical professionalism over time and elaborate on ways in which professionalism has become increasingly nuanced and a complex system of competing types of professionalism [68].

The traditional model of leadership in health care is described as outdated, paternalistic, and placing power and authority in physicians [86], aligning with Hafferty and Castellani’s professional dominance [68]. Conversely, a complexity leadership theory is premised on leadership as co-constructed and emergent, distinguishing between leadership and leaders, and a leadership that sits outside formal leadership positions [65]. Seven elements of complex adaptive systems theory are used to comprise a learning model that recognises the needs of a system, clarifies the need for change, and mobilises self-organising behaviour [86]. How interprofessional healthcare teams enact leadership at a micro level is explored, highlighting both traditional professional hierarchies and the complex emergence of leadership in healthcare, arguing for a shift to a distributed model of leadership [65].

### Learning for systems, learning about systems, and learning in systems

The reviewed literature acknowledges the connection between complexity and systems thinking from two angles: learning for systems through interprofessional learning (IPL) and simulation-based education (SBE) and learning about systems. IPL and SBE is well attended to in the medical education more broadly, the notion of learning about systems is a more recent addition and notably aligned with complexity. Learning environments are also examined with a focus on the systems in which medical education occurs.

There is rich theoretical discussion regarding the relevance of complexity principles to IPL [37, 49, 78], and examples of complexity being applied empirically to IPL activities were plentiful [28, 39, 40, 71, 77, 91, 96, 101, 105]. With the intensely collaborative nature of medicine, dominant individually focused theories are not fit for purpose, and socio-cultural theories offer good fit in dynamic often high-risk contexts such as teamwork [29]. Complexity allows the social and cultural aspects of SBE to come the surface [35] and a reconceptualization of simulation as a pedagogy that is truly innovative, integrative and interprofessional [51]. It is proposed that IPL focus less on individual knowledge and competencies and more on relationship competencies [69], social interactions, cultures and settings, relationships to material elements, documentation methods and diagnostic technologies [78]. Complexity is employed to understand and support the dynamics of professional collaborative learning [49].

Across several publications, Cooper and colleagues describe how complexity has acted as a theoretical framework for the development and evaluation of IPL with its focus on connectivity, diversity, self-organisation and emergence [37–40]. Complexity allows a move from a focus on the individual and how they learn, to a focus on the health team, health system and environment [91]. By providing IPL facilitators with a complexity perspective, they were more confident integrating key complexity principles [77]. Other studies have used complexity informed approaches to design and evaluate IPL activities [39, 40, 71, 96, 101, 105] and in doing so they acknowledge the intensely social nature of the activities involved [71], that student learning is an emergent and constructed process with a need to allow direction to arise through different approaches[39], and that complexity emphasises the dynamic relationship between individuals and their physical and social environments [101]. It is proposed that the use of complexity may not lie far from what educators are already doing, however theory is useful in exploring the messiness within SBE and IPL [66, 75].

Individualistic aspects of the culture of medicine mean a systems-based approach can be unfamiliar to physicians, however an understanding of complexity could lead to an appreciation of the importance of systems [59]. The introduction of systems thinking and complexity informed concepts to the curriculum is argued for to address the challenges of the complex healthcare environment [93, 99]. IPL is a method for preparing students for systems thinking [34] and introducing complexity concepts into graduate and professional education is necessary to drive system changes that address current fragmentation [46, 61, 83]. Across a number of papers, Gonzalo and colleagues argue for the integration of health systems science, alongside basic and clinical sciences in pre-registration medical education [60, 62, 120], with acknowledgement that there are challenges [63].

The multiple environments and contexts where medical training occurs influence learning, teaching and practice of medicine, making complexity a relevant theory for the context of education (physical and situational) [47]. Informal and incidental learning in clinical environments is examined in the context of the COVID-19 Pandemic using a complexity framework [85]. Small group problem-based learning viewed with a complexity lens identifies the learning groups as complex adaptive systems with fuzzy boundaries, whereby if dynamics change, tasks and resources are reconfigured [79]. The principles of non-linearity and co-evolution demonstrate that for teaching hospitals, education interacts and evolves with clinical care [103]. Complexity is also a useful lens when considering the implementation of distributed education, outside tertiary health care [104].

## Discussion

This review explores the narratives and themes that are occurring in the nexus of complexity and medical education into an integrated understanding of its relevance. The review identified 83 articles that clearly and overtly incorporated complexity as a theoretical lens. Authors both rationalized and utilized complexity as a theoretical lens across a range of medical education contexts, with some cautioning an over-enthusiasm to the extent that other useful theories are neglected [49, 84]. Understanding that there are multiple orientations to complexity was crucial in ensuring its correct appropriation [42]. Earlier, the appearance of complexity was largely through theoretical and conceptual papers, however in the latter years its use as a theoretical framework in empirical research becomes apparent. The literature demonstrates that complexity is established in the medical education literature, however it is not yet widespread. Recurrently, authors noted individualistic aspects of the culture of medicine, and that doctors are more comfortable with reductionist epistemologies, citing these as both barriers and motivations for the introduction of complexity as a theory for medical education.

Despite calls for the use of theory in medical education research for over 20 years [121] the community is slow to adopt [122]. However, attention to the importance of theory in medical education and medical education research is growing [123–125]. Emerging approaches in medical education such as programmatic assessment [126], competency-based education [127] and interprofessional simulation-based learning [128] to name a few, inherently involve systems. Complexity therefore is a relevant theoretical lens by which to develop, understand and evaluate such pedagogies, which are increasingly important in preparing doctors for practice the complex chronic disease era of the 21^st^ Century [129]. This review focusses on one theory, however it was apparent in the literature that there are many overlapping theories that can be used concurrently, consistent with the nature of complexity.

This review has several limitations. Primarily it was difficult to clearly demarcate the criteria for inclusion, meaning that reproduction of the article selection may be difficult. Use of the term complexity is pervasive in medicine and medical education; a judgment was necessary to determine whether there was genuine engagement with complexity as a science or theory and a different researcher may have included an expanded or restricted selection of studies. While this is a limitation, the process’s integrity is supported by the knowledge that the reviewer had been examining this body of literature iteratively over 7 years and developed an understanding of the ways in which complexity is adopted more broadly. The decision to include or exclude was clear to the reviewer in most cases. Having a single reviewer determine inclusion is also a potential limitation, however given the nature of this review was appropriate for pragmatic reasons. Further, as with qualitative research, the synthesis and interpretation of the text will be influenced by the researchers’ experiences and expertise. This can be seen as a strength and/or limitation. The heterogeneity of the papers included made integration difficult; any attempt at over-simplification would be counter to the purpose of the review. However, a conceptual model (Fig. 2) that was created – possibly at the risk of simplification – does allow an overview of the breadth of the use of complexity in the medical education literature. Strengths of the review lie in application of the six guiding principles of a meta-narrative review [23], even though the review was not able to be constructed as a meta-narrative review due to the nature of the research question.

## Conclusion

The development of a conceptualization and mixed narrative and thematic review of the use of complexity in medical education has explicated how complexity can aid development of the field and allows researchers to determine the potential for application of complexity as a theoretical framework.

## Supplementary Information

**Additional file 1.** Guiding principles for the review.

**Additional file 2.** List of publications mapped to themes, type of publication and type of engagement with complexity.

## Data Availability

The datasets used and/or analysed during the current study available from the corresponding author on reasonable request. Not applicable.

## Abbreviations

IPL: Interprofessional learning
SBE: Simulation Based Education

## References

[1] Swanwick T, Buckley G, Swanick T (2013). Introduction: Understanding Medical Education. Understanding Medical Education: Evidence, Theory and Practice.

[2] Custers EJFM, Ten Cate O (2018). The history of medical education in Europe and the United States, with respect to time and proficiency. Acad Med.

[3] Geffen L (2014). A brief history of medical education and training in Australia. Med J Aust.

[4] Irby DM, Cooke M, O'Brien BC (2010). Calls for reform of medical education by the Carnegie Foundation for the Advancement of Teaching: 1910 and 2010. Acad Med.

[5] Waldrop MM (1993). Complexity: The emerging science at the edge of order and chaos.

[6] Byrne D (2001). Complexity Theory and the Social Sciences: An Introduction.

[7] Byrne D, Callaghan G (2014). Complexity Theory and the Social Sciences: The state of the art.

[8] Castellani B, Hafferty FW (2009). Sociology and Complexity Science: A New Field of Inquiry.

[9] Begun JW, Zimmerman B, Dooley K, Mick SS, Wyttenbach ME (2003). Health Care Organizations as Complex Adaptive Systems. Advances in Health Care Organization Theory (1 ed).

[10] Martínez-García M, Hernández-Lemus E (2013). Health Systems as Complex Systems. Ame J Oper Res.

[11] Castellani B, Gerrtis L. 2021 Map of the Complexity Sciences Cleveland Ohio: Art and Science Factory; 2021 [Available from: https://www.art-sciencefactory.com/complexity-map_feb09.html].

[12] Morin E, Gershenson C, Aerts D, Edmonds B (2007). Restricted Complexity, General Complexity. Worldviews, science and us: Philosophy and complexity.

[13] Manson SM (2001). Simplifying complexity: a review of complexity theory. Geoforum.

[14] Castellani B. Brian Castellani on the Complexity Sciences: Sage Publications; 2014 [Available from: https://www.theoryculturesociety.org/blog/brian-castellani-on-the-complexity-sciences].

[15] Alhadeff-Jones M, Taylor EW, Cranton P (2012). Transformative Learning and the Challenges of Complexity. Handbook of Transformative Learning: Theory, Research and Practice.

[16] Byrne D (2014). Thoughts on a pedagogy of complexity. Complicity: An International Journal of Complexity and Education.

[17] Davis B, Sumara D (2009). Complexity as a theory of education. Transnational Curriculum Inquiry.

[18] Davis B, Sumara D (2010). If things were simple . . .’: complexity in education. J Eval Clin Pract.

[19] Doll WE (2008). Complexity and the culture of curriculum. Educ Philos Theory.

[20] Jorg T (2017). On reinventing education in the age of complexity: a Vygotsky-inspired generative complexity approach. Complicity: An International Journal of Complexity and Education.

[21] Mason M (2008). Complexity theory and the philosophy of education. Educ Philos Theory.

[22] Greenhalgh T, Hurwitz B, Greenhalgh T (2004). Meta-narrative mapping: a new approach to the systematic review of complex evidence. Narrative Research in Health and Illness.

[23] Greenhalgh T, Robert G, Macfarlane F, Bate P, Kyriakidou O, Peacock R (2005). Storylines of research in diffusion of innovation: a meta-narrative approach to systematic review. Soc Sci Med.

[24] Centre for Reviews and Dissemination. Prospero: International prospective register of systematic reviews University of York, York, UK: National Institute for Health Research; 2022 [Available from: https://www.crd.york.ac.uk/PROSPERO/].

[25] Aron DC (2017). Developing a complex systems perspective for medical education to facilitate the integration of basic science and clinical medicine. J Eval Clin Pract.

[26] Arrow H, Henry KB (2010). Using complexity to promote group learning in health care. J Eval Clin Pract.

[27] Batt AM, Williams B, Brydges M, Leyenaar M, Tavares W (2021). New ways of seeing: supplementing existing competency framework development guidelines with systems thinking. Adv Health Sci Educ.

[28] Berger S, Whelan B, Mahler C, Szecsenyi J, Krug K (2019). Encountering complexity in collaborative learning activities: an exploratory case study with undergraduate health professionals. J Interprof Care.

[29] Bleakley A (2006). Broadening conceptions of learning in medical education: the message from teamworking. Med Educ.

[30] Bleakley A (2010). Blunting Occam's razor: aligning medical education with studies of complexity. J Eval Clin Pract.

[31] Bleakley A (2012). The curriculum is dead! Long live the curriculum! Designing an undergraduate medicine and surgery curriculum for the future. Med Teach.

[32] Bleakley A, Lajoi S, Steinert Y (2014). Team Process and Complexity Theory: Blunting Occam's Razor. Patient-Centred Medicine in Transition The Heart of the Matter.

[33] Bleakley A, Cleland J, Cleland J, Durning SJ (2015). Sticking with Messy Realities: How 'Thinking with Complexity' can Inform Healthcare Education Research. Researching Medical Eduction.

[34] Clark K, Hoffman A (2019). Educating healthcare students: strategies to teach systems thinking to prepare new healthcare graduates. J Prof Nurs.

[35] Cleland J, Walker KG, Gale M, Nicol LG (2016). Simulation-based education: understanding the socio-cultural complexity of a surgical training 'boot camp'. Med Educ.

[36] Cleland JA, Patterson F, Hanson MD (2018). Thinking of selection and widening access as complex and wicked problems. Med Educ.

[37] Cooper H, Braye S, Geyer R (2004). Complexity and interprofessional education. Learn Health Soc Care.

[38] Cooper H, Geyer R (2008). Using 'complexity' for improving educational research in health care. Soc Sci Med.

[39] Cooper H, Spencer-Dawe E (2006). Involving service users in interprofessional education narrowing the gap between theory and practice. J Interprof Care.

[40] Cooper H, Spencer-Dawe E, McLean E (2005). Beginning the process of teamwork: design, implementation and evaluation of an inter-professional education intervention for first year undergraduate students. J Interprof Care.

[41] Cristancho S (2014). Fewer themes, more stories: shall we consider alternative ways for representing complexity well?. Perspect Med Educ.

[42] Cristancho S, Field E, Lingard L (2019). What is the state of complexity science in medical education research?. Med Educ.

[43] Cristancho SM, Helmich E (2019). Rich pictures: a companion method for qualitative research in medical education. Med Educ.

[44] Dickey J, Girard DE, Geheb MA, Cassel CK (2004). Using systems-based practice to integrate education and clinical services. Med Teach.

[45] Doll WE, Trueit D (2010). Complexity and the health care professions. J Eval Clin Pract.

[46] Dornan T, Lee C, Findlay-White F, Gillespie H, Conn R (2021). Acting wisely in complex clinical situations: ‘mutual safety’ for clinicians as well as patients. Med Teach.

[47] Durning SJ, Artino AR, Pangaro LN, van der Vleuten C, Schuwirth L (2010). Perspective: redefining context in the clinical encounter: implications for research and training in medical education. Acad Med.

[48] Durning SJ, Lubarsky S, Torre D, Dory V, Holmboe E (2015). Considering, “nonlinearity” across the continuum in medical education assessment: supporting theory, practice, and future research directions. J Contin Educ Health Prof.

[49] Fenwick T (2012). Complexity science and professional learning for collaboration: a critical reconsideration of possibilities and limitations. J Educ Work.

[50] Fenwick T (2014). Sociomateriality in medical practice and learning: attuning to what matters. Med Educ.

[51] Fenwick T, Dahlgren MA (2015). Towards socio-material approaches in simulation-based education: lessons from complexity theory. Med Educ.

[52] Fernandez N, Audetat MC (2019). Faculty development program evaluation: a need to embrace complexity. Adv Med Educ Pract.

[53] Frank JR, Snell LS, Oswald A, Hauer KE, for the International CBME Collaborators (2021). Further on the journey in a complex adaptive system: elaborating CBME. Med Teach..

[54] Fraser SW, Greenhalgh T (2001). Coping with complexity: educating for capability. BMJ.

[55] Frye AW, Hemmer PA (2012). Program evaluation models and related theories: AMEE Guide No. 67 -complexity. Med Teach.

[56] Glynn LG, Scully R (2010). The edge of chaos: reductionism in healthcare and health professional training. Int J Clin Pract.

[57] Goldman EF, Mintz ML (2017). Using concepts from complexity science to accelerate curricular revision. Innov High Educ.

[58] Goldszmidt M (2017). When I say … Sociomateriality. Med Educ.

[59] Gonnering RS (2010). Complexity theory and the "puzzling" competencies: systems-based practice and practice-based learning explored. J Surg Educ.

[60] Gonzalo JD, Caverzagie KJ, Hawkins RE, Lawson L, Wolpaw DR, Chang A (2017). Concerns and responses for integrating health systems science Into medical education. Acad Med.

[61] Gonzalo JD, Wolpaw DR, Cooney R, Mazotti L, Reilly JB, Wolpaw T (2022). Evolving the systems-based practice competency in graduate medical education to meet patient needs in the 21st-century health care system. Acad Med.

[62] Gonzalo JD, Haidet P, Papp KK, Wolpaw DR, Moser E, Wittenstein RD, Wolpaw T (2017). Educating for the 21st-century health care system: an interdependent framework of basic, clinical, and systems sciences. Acad Med.

[63] Gonzalo JD, Ogrinc G (2019). Health systems science: the "broccoli" of undergraduate medical education. Acad Med.

[64] Gordon L, Cleland JA (2021). Change is never easy: how management theories can help operationalise change in medical education. Med Educ.

[65] Gordon L, Rees C, Ker J, Cleland J (2017). Using video-reflexive ethnography to capture the complexity of leadership enactment in the healthcare workplace. Adv Health Sci Educ.

[66] Gormley GJ, Fenwick T (2016). Learning to manage complexity through simulation: students' challenges and possible strategies. Perspect Med Educ.

[67] Hafferty F, Levinson D (2008). Moving beyond nostalgia and motives: towards a complexity science view of medical professionalism. Perspect Biol Med.

[68] Hafferty FW, Castellani B (2010). The increasing complexities of professionalism. Acad Med.

[69] Hall P, Weaver L, Grassau PA (2013). Theories, relationships and interprofessionalism: learning to weave. J Interprof Care.

[70] Hill F, Stephens C (2005). Building leadership capacity in medical education: developing the potential of course coordinators. Med Teach.

[71] Jorm C, Nisbet G, Roberts C, Gordon C, Gentilcore S, Chen TF (2016). Using complexity theory to develop a student-directed interprofessional learning activity for 1220 healthcare students. BMC Med Educ.

[72] Jorm C, Roberts C (2018). Using complexity theory to guide medical school evaluations. Acad Med.

[73] Khanna P, Roberts C, Lane AS (2021). Designing health professional education curricula using systems thinking perspectives. BMC Med Educ.

[74] Lim D, Schoo A, Lawn S, Litt J (2019). Embedding and sustaining motivational interviewing in clinical environments: a concurrent iterative mixed methods study. BMC Med Educ.

[75] Ma IWA (2015). Embracing complexity: taking the messiness in simulation-based training one step further. Med Educ.

[76] McLellan L, Yardley S, Norris B, de Bruin A, Tully M, Dornan T (2015). Preparing to prescribe: How do clerkship students learn in the midst of complexity?. Adv in Health Sci Educ.

[77] McMurtry A (2010). Complexity, collective learning and the education of interprofessional health teams: insights from a university-level course. J Interprof Care.

[78] McMurtry A, Rohse S, Kilgour KN (2016). Socio-material perspectives on interprofessional team and collaborative learning. Med Educ.

[79] Mennin S (2007). Small-group problem-based learning as a complex adaptive system. Teach Teach Educ.

[80] Mennin S (2010). Complexity and health professions education. J Eval Clin Pract.

[81] Mennin S (2010). Self-organisation, integration and curriculum in the complex world of medical education. Med Educ.

[82] Mennin SP, Sturmberg JP, Martin CM (2013). Health Professions Education: Complexity, Teaching, and Learning. Handbook of Systems and Complexity in Health.

[83] Norman C (2013). Teaching systems thinking and complexity theory in health sciences. J Eval Clin Pract.

[84] Norman G (2011). Chaos, complexity and complicatedness: lessons from rocket science. Med Educ.

[85] Papanagnou D, Watkins KE, Lundgren H, Alcid GA, Ziring D, Marsick VJ (2022). Informal and incidental learning in the clinical learning environment: learning through complexity and uncertainty during COVID-19. Acad Med.

[86] Prather SE, Jones DN (2003). Physician leadership: influence on practice-based learning and improvement. J Contin Educ Health Prof.

[87] Rees C, Richards L (2004). Outcomes-based education versus coping with complexity: should we be educating for capability?. Med Educ.

[88] Regehr G (2010). It’s NOT rocket science: rethinking our metaphors for research in health professions education. Med Educ.

[89] Regehr G (2011). Highway spotters and traffic controllers: further reflections on complexity. Med Educ.

[90] Rojas D, Grierson L, Mylopoulos M, Trbovich P, Bagli D, Brydges R (2018). How can systems engineering inform the methods of programme evaluation in health professions education?. Med Educ.

[91] Sargeant J (2009). Theories to aid understanding and implementation of interprofessional education. J Contin Educ Health Prof.

[92] Sargeant J, Wong BM, Campbell CM (2018). CPD of the future: a partnership between quality improvement and competency-based education. Med Educ.

[93] Sawyer NT, Danielson A, Johl K, Williams DM (2021). Introduction to health systems science: experiential learning through patient interviews in the emergency department. AEM Education and Training.

[94] Schoo A, Kumar K (2018). The clinical educator and complexity: a review. Clin Teach.

[95] Smith CS, Francovich C, Morris M, Hill W, Langlois-Winkle F, Rupper R, Roth C (2010). Toward an ecological perspective of resident teaching clinic. Adv in Health Sci Educ.

[96] Solomon P, Risdon C (2014). A process oriented approach to promoting collaborative practice: incorporating complexity methods. Med Teach.

[97] Sturmberg J, Hinchy J (2010). Borderline competence – from a complexity perspective: conceptualization and implementation for certifying examinations. J Eval Clin Pract.

[98] Sturmberg J, Martin C (2008). Knowing – in Medicine. J Eval Clin Pract.

[99] Swanson RC, Cattaneo A, Bradley E, Chunharas S, Atun R, Abbas KM, Katsaliaki K (2012). Rethinking health systems strengthening: key systems thinking tools and strategies for transformational change. Health Policy Plan.

[100] Talbot M (2004). Good wine may need to mature: a critique of accelerated higher specialist training. Evidence from cognitive neuroscience. Med Educ.

[101] Thompson DS, Abourbih J, Carter L, Adams-Carpino G, Berry S, Graves LE, Ranger NJ (2018). Views from the field: medical student experiences and perceptions of interprofessional learning and collaboration in rural settings. J Interprof Care.

[102] Torre D, Schuwirth L, Van der Vleuten C, Heeneman S (2022). An international study on the implementation of programmatic assessment: understanding challenges and exploring solutions. Med Teach.

[103] van Rossum TR, Scheele F, Scherpbier AJ, Sluiter HE, Heyligers IC (2016). Dealing with the complex dynamics of teaching hospitals. BMC Med Educ.

[104] Van Schalkwyk SC, Couper ID, Blitz J, De Villiers MR (2020). A framework for distributed health professions training: using participatory action research to build consensus. BMC Med Educ.

[105] Weaver L, McMurtry A, Conklin J, Brajtman S, Hall P (2011). Harnessing complexity science for interprofessional education development: a case study. J Res Interprof Pract Educ.

[106] Woodruff JN (2019). Accounting for complexity in medical education: a model of adaptive behaviour in medicine. Med Educ.

[107] Federman DD (1990). The education of medical students: sounds, alarums, and excursions. Acad Med.

[108] Plsek P, Greenhalgh T (2001). The challenge of complexity in health care. BMJ.

[109] Plsek P, Wilson T (2001). Complexity, leadership, and management in healthcare organisations. BMJ.

[110] Wilson T, Holt T, Greenhalgh T (2001). Complexity and clinical care. BMJ.

[111] Flexner A. Medical Education in the United States and Canada. A report to the Carnegie Foundation for the Advancement of Teaching. New York City; 1910. Available from: http://archive.carnegiefoundation.org/pdfs/elibrary/Carnegie_Flexner_Report.pdf. PMC2567554 12163926

[112] Greenhalgh T, Plsek P, Wilson T, Fraser S, Holt T (2010). Response to 'The appropriation of complexity theory in health care'. J Health Serv Res Policy.

[113] Paley J (2010). The appropriation of complexity theory in health care. J Health Serv Res Policy.

[114] Reid I (2002). Complexity science: Let them eat complexity: the emperor's new toolkit. BMJ.

[115] Paley J (2007). Complex adaptive systems and nursing. Nurs Inq.

[116] Orlikowski WJ (2007). Sociomaterial practices: exploring technology at work. Organ Stud.

[117] Fenwick T, Nerland M, Jensen K (2012). Sociomaterial approaches to conceptualising professional learning and practice. J Educ Work.

[118] Duignan B. Occam’s razor: Encyclopedia Britannica; 2021 [Available from: https://www.britannica.com/topic/Occams-razor].

[119] Cleary TJ, Durning SJ, Artino ARJ (2016). Microanalytic assessment of self-regulated learning during clinical reasoning tasks: recent developments and next steps. Acad Med.

[120] Gonzalo JD, Ahluwalia A, Hamilton M, Wolf H, Wolpaw DR, Thompson BM. Aligning education with health care transformation: identifying a shared mental model of "new" faculty competencies for academic faculty. Acad Med. 2017. 10.1097/ACM.0000000000001895 28991850

[121] Prideaux D, Spencer J (2000). On theory in medical education. Med Educ.

[122] Rees CE, Monrouxe LV (2010). Theory in medical education research: how do we get there?. Med Educ.

[123] Varpio L, Ellaway RH (2021). Shaping our worldviews: a conversation about and of theory. Adv Health Sci Educ.

[124] Mukhalalati B, Elshami S, Eljaam M, Hussain FN, Bishawi AH (2022). Applications of social theories of learning in health professions education programs: a scoping review. Front Med.

[125] Kaufman DM. Teaching and Learning in Medical Education: How Theory Can Inform Practice. In: Understanding Medical Education: Evidence, Theory, and Practice. 2018: 37–69.

[126] van der Vleuten C, Lindemann I, Schmidt L (2018). Programmatic assessment: the process, rationale and evidence for modern evaluation approaches in medical education. Med J Aust.

[127] ten Cate O, Billett S (2014). Competency-based medical education: origins, perspectives and potentialities. Med Educ.

[128] Masiello I (2012). Why simulation-based team training has not been used effectively and what can be done about it. Adv Health Sci Educ Theory Pract.

[129] Lucey C (2013). Medical education: Part of the problem and part of the solution. JAMA Intern Med.

