# Supplementary material for: Examining the nexus between medical education and complexity: a systematic review to inform practice and research
Source: BMC Med Educ. 2023 Jul 5;23:494. doi: 10.1186/s12909-023-04471-2 (PMC10320888; doi:10.1186/s12909-023-04471-2)
Supplement: Supplementary file 1 — Additional file 1. Guiding principles for the review. [file 12909_2023_4471_MOESM1_ESM.docx]

**Guiding principles (based on the guiding principles for a meta-narrative review)**

The following outlines how the guiding principles for a meta-narrative review[1] were addressed.

- Pragmatism: Inclusion and exclusion boundaries were not clearly evident and pragmatic decision were sometimes made about whether to include a publication. The review relied on the developing understanding of the broader literature on complexity by the authors to determine what papers would contribute usefully to the review. For example the publication by Davis and Sumara’s [2] is included because even though it focuses on the context of health professionals education broadly, their publication pioneered the integration of complexity as an educational theory. However, other publications that were not directly relevant to medical education were excluded. Ultimately, pragmatism determined that this was not a meta-narrative review, and the methods were changed accordingly. However, it remained that the guiding principles continued to be relevant.
- Pluralism: As complexity is a relatively nascent theoretical lens for medical education, all publications which truly engaged in complexity (as it relates to a science or theory) were considered for inclusion. Given the empirical use of complexity in medical education research is not yet widespread, all studies which purported to use complexity in their design or analytical approach were included in order to build a picture of how the research body develops over time. However, the nature of this review is to exclude other research traditions, and this is why a meta-narrative review was ultimately not possible. Pluralism in terms of the nature of the publications included remains.
- Historicity: The most significant narrative in this review is that of describing the evolution of the use of complexity in medical education. The evolution of complexity across many systems, including education and health is also acknowledged and has been described, this review examines and embraces the historicity of complexity and its interaction with medical education.
- Contestation: The use of complexity in medical education and medical education research is not established, and there is known contestation about its application in healthcare systems and social systems. Similar challenges to its legitimacy in medical education have been identified as a narrative and papers were specifically sought to ensure that this was not excluded.
- Reflexivity: The team continually reflected on the identified literature, what the nature of the literature was telling us, and what the content was revealing. The review was originally conceived as a meta-narrative review. Its evolution identified that this review does not sit comfortably with any well described methodology, and we had to pragmatically deal with this. Reflecting on the findings enabled us to determine that the value in this review would be a combination of narrative and thematic description to inform medical educators as to the state of complexity in medical education broadly and the potential uses more specifically.
- Peer review: This literature review was conducted in the context of the PhD studies of the primary author and peer reviewed by supervisors. Once submitted as a potential publication the journal peer reviewers played a significant role in the final version of this paper. Although anonymous, it was clear that they had significant experience in this space and understood the value and purpose of the review. The reviewers provided a level of peer feedback which led to significant re-framing and revision of the manuscript that truly impacted on the quality of the final manuscript.

1. Greenhalgh T, Robert G, Macfarlane F, Bate P, Kyriakidou O, Peacock R. Storylines of research in diffusion of innovation: a meta-narrative approach to systematic review. Soc Sci Med. 2005; 61(2):417-430.

2. Davis B, Sumara D. ‘If things were simple . . .’: complexity in education. J Eval Clin Pract. 2010; 16(4):856-860.
